# THE LONGITUDINAL EFFECT OF FINANCIAL DIFFICULTY ON CAREGIVER DEPRESSION

**DOI:** 10.1093/geroni/igac059.2106

**Published:** 2022-12-20

**Authors:** Susanna Mage, Kathleen Wilber, Donna Benton

**Affiliations:** University of Southern California, Leonard Davis School of Gerontology, Los Angeles, California, United States; University of Southern California, Los Angeles, California, United States; University of Southern California, Los Angeles, California, United States

## Abstract

Informal caregivers are the backbone of the long-term healthcare system in the United States and crucial in supporting the rapidly increasing aging population. Previous studies have illustrated the negative effects that the caregiving role can have on caregiver mental health, but few studies have examined the singular role of experiencing financial difficulty on caregiver mental health. The aim of this study was twofold: to measure how financial difficulty in 2015 correlates with caregiver depression in 2015, and to examine whether financial difficulty in 2015 can predict caregiver depression in 2017. We used two-wave panel data from the National Study on Caregiving (N=1,125) to conduct both regression and lagged dependent variable (LDV) regression analyses to investigate the cross-sectional and longitudinal effect of financial difficulty on caregiver depression, respectively. Caregiver depression was measured using the 2-item version of the Patient Health Questionnaire-9 (PHQ-2), and financial difficulty was self-reported. Results show that caregivers who did not experience financial difficulty in 2015 had 0.46 times the odds of not being depressed (CI 0.30-0.70, p-value= 0.00) in 2015, compared to those who noted that they did experience financial difficulty. In performing a LDV analysis, outcomes showed that financial difficulty in 2015 was significantly associated with caregiver depression in 2017 (p-value< 0.05), implying that current reported financial difficulty may influence caregiver depression two years later. Conclusions from this work provide support for the development of financial interventions for caregivers experiencing financial difficulty that could play a role in alleviating their depressive symptoms.

